# Research on UAV Three-Phase Transmission Line Tracking and Localization Method Based on Electric Field Sensor Array

**DOI:** 10.3390/s21248400

**Published:** 2021-12-16

**Authors:** Chunguang Suo, Jiawen Zhao, Wenbin Zhang, Peng Li, Rujin Huang, Junyu Zhu, Xiangyu Tan

**Affiliations:** 1 College of Science, Kunming University of Science and Technology, Kunming 650504, China; suochunguang@kust.edu.cn (C.S.); zhaojiawen@stu.kust.edu.cn (J.Z.); huangrujin@stu.kust.edu.cn (R.H.); zhujunyu@stu.kust.edu.cn (J.Z.); 2 College of Mechanical and Electrical Engineering, Kunming University of Science and Technology, Kunming 650504, China; lipeng@stu.kust.edu.cn; 3 Electric Power Research Institute, Yunnan Power Grid Co., Ltd., Kunming 650217, China; 2040464@163.com

**Keywords:** transmission lines, tracking and localization, UAV, electric field sensor array

## Abstract

The tracking and positioning of transmission lines is a key element for UAVs (Unmanned Aerial Vehicles) to achieve autonomous inspection of transmission lines. Current methods are vulnerable to weather and environmental factors, have high costs, and have difficulties in data processing. Therefore, this paper proposes a transmission line tracking and localization method based on the electric field sensor array, which calculates the current UAV’s heading angle deflection angle, the distance between the transmission line and the UAV, and the elevation angle, providing a new idea to solve the problem of UAV inspection of transmission lines. At the same time, the electric field distribution of different arrangements of three-phase transmission lines was analyzed using COMSOL to determine the flight area of the UAV. By comparing the electric field distribution of the UAV flight area and single-phase transmission lines, it was verified that the current method is also applicable in the three-phase transmission line scenario, and it was further verified that the sensor array used can sense the change of the UAV position in the flight area, indicating that the electric field sensor array can realize the transmission line tracking and localization of transmission lines. The experimental results showed that, in the three-phase transmission line scenario, when the sensor array moves along the transmission straight wire, the maximum absolute error of the heading angle deflection angle calculated according to this method was 8.2°, the maximum absolute error of the distance between the array and the transmission line was 19.3 cm, and the maximum absolute error of the elevation angle was 11.37°; the error was within a reasonable range and can be used for the UAV to realize autonomous inspection.

## 1. Introduction

With the continuous development of the power system, the scale of high-voltage overhead power lines has grown unprecedentedly, and in recent years drones have been widely used in the inspection of transmission lines [1]. Due to the low efficiency of manual drone inspection and the limited vision of the operator, it is difficult to visually inspect the distance between the drone and transmission lines and obstacles, once the risk of operational errors, which will cause the drone to crash and damage the transmission lines. Therefore, the technology of automatic inspection of transmission lines by drones has become a hot topic of research nowadays.

Automatic inspection of transmission lines by drones brings efficiency gains and makes up for the shortcomings of manually operated drones, but it also brings many challenges. Reference [2] proposed a tower and conductor tracking algorithm that uses GPS information to quickly calculate the distance between two points on the premise that the current position of the UAV (Unmanned Aerial Vehicle) and the GPS information of the tower in the inspection line are known. However, the accuracy of GPS range was low, and a large number of base stations must be built to improve the accuracy, and the cost of GPS itself was not high, but the cost of building base stations was higher. Reference [3] proposed a vision-based transmission line inspection method for UAVs, which has the problem that it is easily affected by the environment and cannot achieve autonomous navigation when there is foggy weather to see transmission lines clearly. Reference [4] proposed a LiDAR-based power line detection method, LiDAR through the rapid acquisition of line channel, high-precision, three-dimensional point cloud data to plan the flight heading to achieve autonomous inspection, for which a large amount of data are required to extract the power line and tower information from the disordered laser point cloud, and the detection efficiency was low. References [5,6] shows that the carrier phase difference technology (real-time kinematic, RTK) can also realize the autonomous cruise of UAVs, and its positioning accuracy can reach the centimeter level. However, RTK technology needs to establish a reference base station or have a stable and fast network coverage to provide a virtual base station. Additionally, the overhead lines are widely distributed and most of them are in populated rare areas. Therefore, it is difficult to achieve full coverage of RTK base stations.

The research on applying electromagnetic field information in the space around overhead transmission lines to UAV inspection of transmission lines is relatively rare, and the current relevant research in this area is divided into three main areas: (1) research based on magnetic field information; (2) research based on electric field information; and (3) research based on a combination of electric and magnetic field information.

Regarding the application of magnetic field information in UAVs, scholars have done the following studies. Reference [7] calculates the position of the UAV relative to the transmission line by building an array of magnetic field sensors. Reference [8] estimates the flight trajectory of the UAV based on the magnetic field information around the transmission line using an extended Kalman filter algorithm. Reference [9] designed a dynamic electromagnetic positioning system in which three quasi-static electromagnetic generators were placed in the UAV landing zone. A magnetic field sensor was installed on the UAV, and the coordinates and azimuth of the UAV were measured during the UAV’s approach to the landing zone to achieve an accurate landing of the UAV. Reference [10] reconstructed the position of the line from the magnetic field data based on the proposed comprehensive algorithm combining the metaheuristic algorithm and the interior point method. Several drawbacks of using magnetic field information are that in the actual transmission line there is a non-linear load effect, the current in the transmission line is unstable, the magnetic field generated by it is also unstable, and there is uncertainty in the calculation results.

Research has also been done on the application of electric field information to UAVs. Reference [11] Evaluates the deviation between the UAV’s position and the preset path based on the electric field measured by the sensor and the theoretical electric field value of the preset path. Reference [12] placed a triangular array of electric field sensors on the ground and detected the flight path of the aircraft over a range of 40 to 500 m by this system. References [13,14] established the fitting relationship between the electric field strength and distance by the data of the measured electric field, and calculated the distance between the UAV and the transmission line based on the fitting relationship. Reference [15] proposed a safe distance diagnosis method for UAVs based on multi-sensor data fusion, which includes the field strength information obtained by the UAV. However, this method does not calculate the distance between the UAV and the transmission line, but only gives a state of whether the UAV is currently safe or not. Reference [16] used electric field sensor arrays to achieve the localization for aerially charged bodies, but the objects targeted by this method differ significantly from transmission lines and cannot be applied to UAVs to achieve automatic inspection. Reference [17] proposed an unmanned aircraft navigation and positioning system based on electric field information. However, the method is the result of considering the three-phase transmission line as a field source, the field intensity distribution under the actual three-phase transmission line is complex, and there are large differences between them. Some ideas based on the application of combined electric and magnetic fields have also been proposed by related scholars. Patent [18] proposes to use the cross product of the magnetic field vector and the electric field vector to obtain the Poynting vector and use the angle between the direction of the Poynting vector and the direction of the current velocity vector to achieve the navigation of the UAV.

In summary, in order to solve the problems of methods based on machine vision, LiDAR, and GPS, this paper proposes a method for tracking and locating transmission lines based on electric field. The main innovation points of this research are as follows:

1. A transmission line localization model based on an array of electric field sensors was studied.
2. The design of an electric field sensor array with integrated transmission line tracking and positioning.
3. A new idea to solve the problem of UAV inspection of transmission lines is provided.

## 2. Theoretical Derivation of Transmission Line Tracking and Positioning

Many studies have shown [19,20] that AC overhead transmission lines have an industrial frequency electric field in the surrounding space, and the electric field strength at the space observation point is directly related to the distance from the transmission line. Additionally, the distance of the electric field propagation of the industrial frequency is also very long, the electric field strength is not affected by the ambient light intensity and ambient temperature, and the adaptability is high. Moreover, the voltage in the actual transmission line is stable and the electric field generated by it is also stable. Therefore, this paper adopted the electric field information to realize the tracking and positioning of transmission lines.

**Figure 1:**
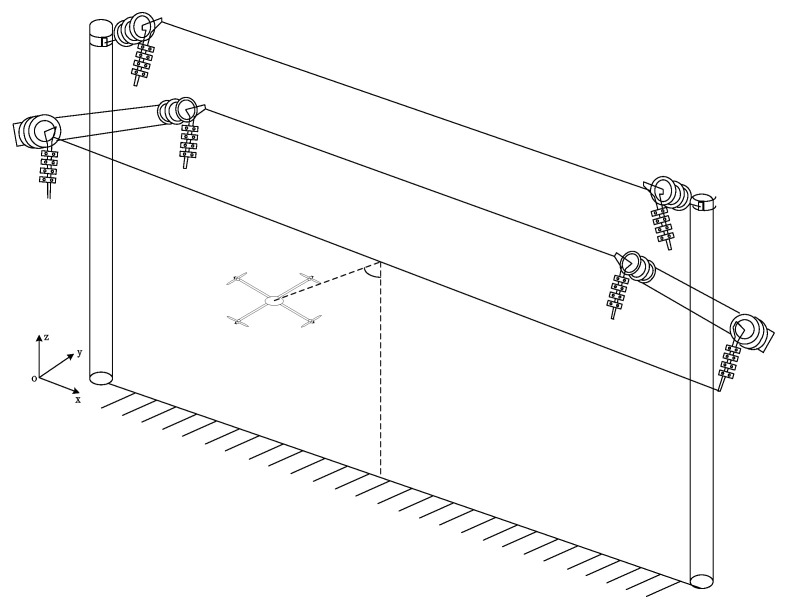
UAV patrol diagram.

As shown in Figure 1 below, to achieve tracking and positioning for transmission lines, the following three parameters need to be calculated based on the electric field information:

(1) The UAV’s heading angle deflection size: The heading angle refers to the angular deviation between the UAV nose and the UAV patrol direction. The UAV realizes the tracking of transmission lines by controlling the deflection size of the heading angle.
(2) Distance of the transmission line relative to the UAV: the distance between the center of the sensor array and the transmission line.
(3) Elevation angle of the transmission line relative to the UAV: The elevation angle reflects the specific orientation of the UAV relative to the transmission line.

Combining the two parameters of (2) and (3), the UAV can determine the location of the transmission line and realize the positioning of the transmission line.

**Figure 2:**
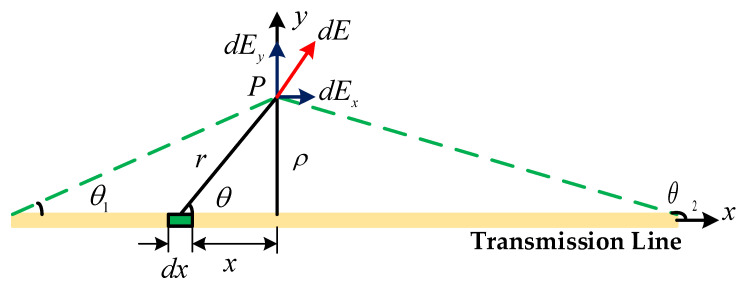
Diagram of transmission line.

As shown in Figure 2, Consider a transmission line as a uniformly charged straight wire of length L and total charge q. Assume that there is a point *P* outside the line, the vertical distance from the straight wire is ρ, and the angle between point *P* and the line at both ends of the line are $\theta_{1}$ and $\theta_{2}$. Let the line charge density be λ and the vacuum dielectric constant be $\varepsilon_{0}$.

Take a charge element:

(1) dq=λdx

According to the formula for the field strength of a point charge, we have:

(2) $$dE_{x}=\frac{\lambda dx}{4\pi \varepsilon_{{}_{0}}r^{2}}\cos\theta$$

(3) $$dE_{y}=\frac{\lambda dx}{4\pi \varepsilon_{0}r^{2}}\sin\theta$$

According to Equations (2) and (3), the field strengths in the x-direction and y-direction can be derived as:

(4) $$E_{x}=\frac{\lambda }{4\pi \varepsilon_{0}\rho }(\sin\theta_{2}-\sin\theta_{1})$$

(5) $$E_{y}=\frac{\lambda }{4\pi \varepsilon_{0}\rho }(\cos\theta_{1}-\cos\theta_{2})$$

Since the size of the UAV itself is much smaller than the length of the transmission line, the transmission line can be considered as an infinitely long straight wire with $\theta_{1}\;$= 0 and $\theta_{2}\;$= π. Then, it also follows that:

(6) $$E_{x}=0$$

(7) $$E_{y}=\frac{\lambda }{2\pi \varepsilon_{0}\rho }$$

(8) $$E=\sqrt{E_{x}{}^{2}+E_{y}{}^{2}}=\frac{\lambda }{2\pi \varepsilon_{0}\rho }$$

It can be known through Equation (8) that if an electric field sensor is placed around a transmission line and the line charge density λ of the transmission line is known, then the distance between the sensor and the transmission line can be calculated from the electric field strength measured by the sensor. However, during the actual inspection of transmission lines by UAVs, this parameter is uncertain, and it is impossible to calculate the distance from the transmission line by one sensor.

**Figure 3:**
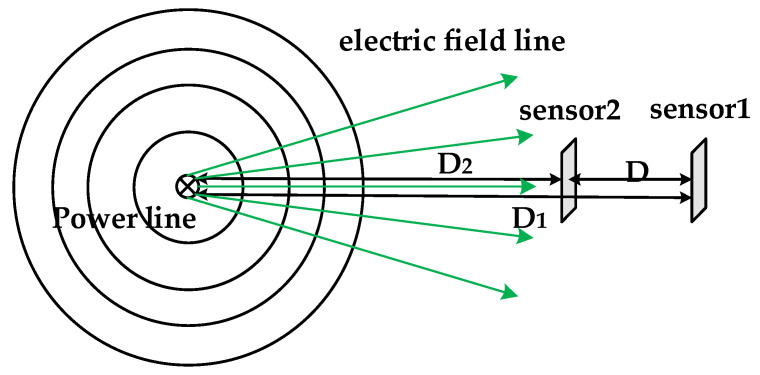
Schematic diagram of the location of the two sensors and the transmission line.

If two sensors are placed, the positions of the two sensors in relation to the transmission line are shown in Figure 3.

Based on Equation (8) and the position relationship between the two sensors, the following two relationships can be obtained:

(9) $$\left\{ \begin{array}{l} \frac{E_{1}}{E_{2}}=\frac{D_{2}}{D_{1}}\\ D_{2}-D_{1}=D \end{array}\right.$$

In the above, the distance *D* between the two sensors is known. Therefore, the distance between the two sensors and the transmission line can also be calculated based on the electric field values measured by the two sensors:

(10) $$\left\{ \begin{array}{l} D_{1}=D\frac{E_{2}}{E_{1}-E_{2}}\\ D_{2}=D\frac{E_{1}}{E_{1}-E_{2}} \end{array}\right.$$

To achieve the positioning of the UAV for the transmission line, in addition to calculating the distance of the UAV from the transmission line, it is also necessary to know the orientation of the transmission line, and the orientation information cannot be calculated based on two sensors. Therefore, it is necessary to place more than two sensors around the transmission line and solve the current sensor position relative to the transmission line based on the position relationship between each sensor.

**Figure 4:**
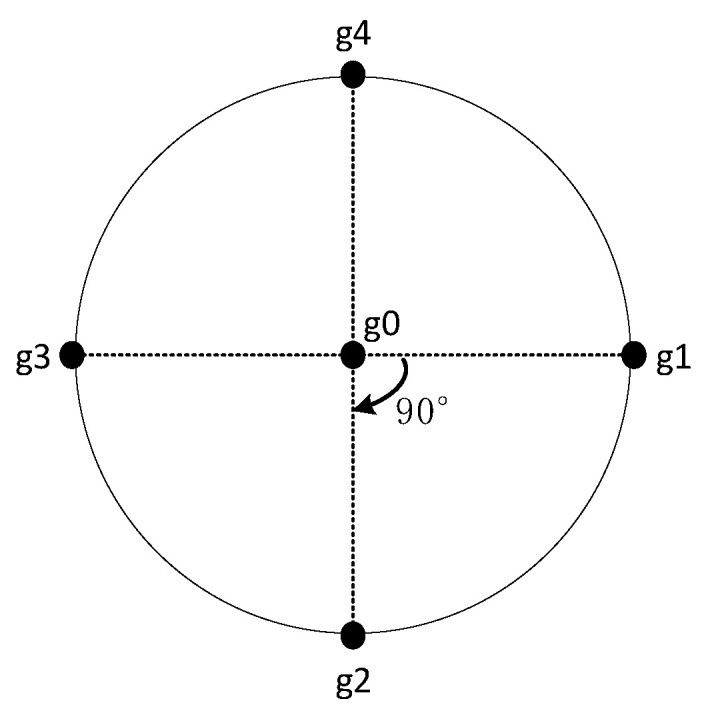
Schematic diagram of the sensor array plane.

Based on this, the sensor array shown in Figure 4 is built. It can be seen that the four sensors, g1, g2, g3, and g4, are evenly distributed on the circular array, and the g0 sensor is located in the center of the circular array.

**Figure 5:**
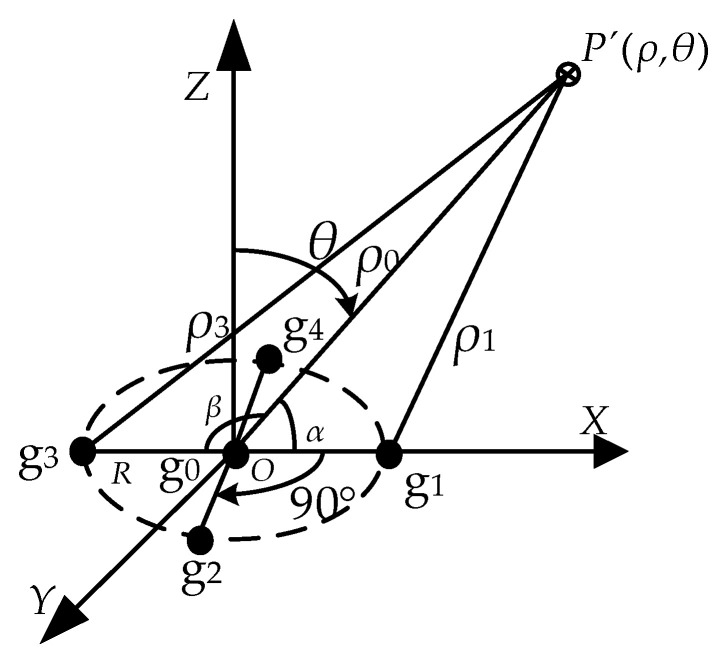
Schematic diagram of the sensor array.

The distance and elevation angle of the drone to the transmission line are based on the values of the three sensors, g3, g0, and g1. Where the three sensors, g3, g0, and g1, are located in a straight line perpendicular to the transmission line, a spatial location of the sensor array and the transmission line is shown schematically in Figure 5.

According to the geometric position relationship between the sensor array and the transmission line in the above figure, in △P’Og1, using the cosine theorem, it is obtained that:

(11) $$R^{2}+\rho_{0}{}^{2}-2R\rho_{0}\cos\alpha =\rho_{1}{}^{2}$$

In △P´g3O, according to the cosine theorem:

(12) $$R^{2}+\rho_{0}{}^{2}-2R\rho_{0}\cos\beta =\rho_{3}{}^{2}$$

According to the induced formula of the triangle, the two equations above can be combined to obtain:

(13) $$\rho_{1}{}^{2}+\rho_{3}{}^{2}-2R^{2}-2\rho_{0}{}^{2}=0$$

In addition, according to Equation (8), then the following relationship can be obtained:

(14) $$\frac{\rho_{0}}{\rho_{1}}=\frac{E_{1}}{E_{0}}=\eta_{1},\frac{\rho_{3}}{\rho_{0}}=\frac{E_{0}}{E_{3}}=\eta_{2},\frac{\rho_{3}}{\rho_{1}}=\frac{E_{1}}{E_{3}}=\eta_{3}$$

According to Equations (13) and (14), it is obtained that:

(15) $$\rho_{0}=\eta_{1}\sqrt{\frac{2R^{2}}{1+\eta_{3}{}^{2}-2\eta_{1}{}^{2}}}$$

(16) $$\rho_{1}=\sqrt{\frac{2R^{2}}{1+\eta_{3}{}^{2}-2\eta_{1}{}^{2}}}$$

The $\rho_{0}$ is the distance of sensor g0 from the transmission line at the center of the sensor array, and $\rho_{1}$ is the distance of sensor g1 from the transmission line.

According to Equations (11), (15) and (16) above, it can be found that:

(17) $$\cos\alpha =\frac{1+\eta_{1}{}^{2}\frac{2}{1+\eta_{3}{}^{2}-2\eta_{1}{}^{2}}-\frac{2}{1+\eta_{3}{}^{2}-2\eta_{1}{}^{2}}}{2\eta_{1}\sqrt{\frac{2}{1+\eta_{3}{}^{2}-2\eta_{1}{}^{2}}}}$$

where α is the elevation angle of the transmission line with respect to the UAV.

**Figure 6:**
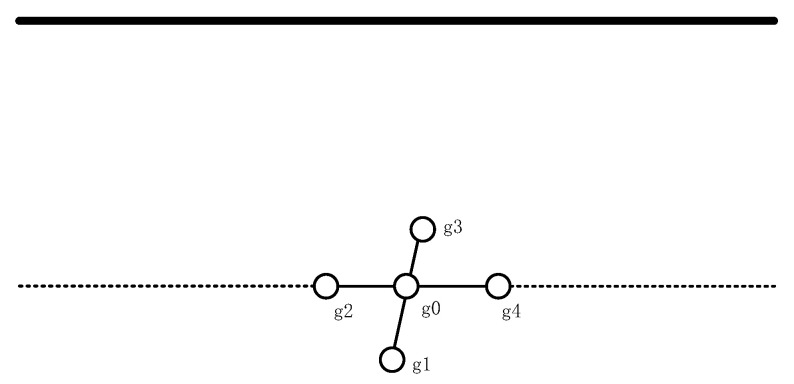
UAV patrol schematic.

Figure 6 is a schematic diagram of the UAV carrying a sensor array for line patrol. When the straight line where the g2, g0, and g4 sensors are located is parallel to the transmission line, the electric field intensity induced by the two sensors is equal. When the straight line where g2, g0, and g4 are located intersects with the transmission line, the electric field intensity induced by the two sensors is not equal. Based on this, the difference between the electric field strengths sensed by the g2 and g4 sensors can be used to control the amount of angular deflection of the UAV as it flies along the transmission line.

(18) $$\Delta \varphi =E_{4}-E_{2}$$

where *E_4_* and *E_2_* are the electric field strengths induced by the two sensors g4 and g2, respectively, and Δφ is the is the deflection size of the UAV heading angle.

**Figure 7:**
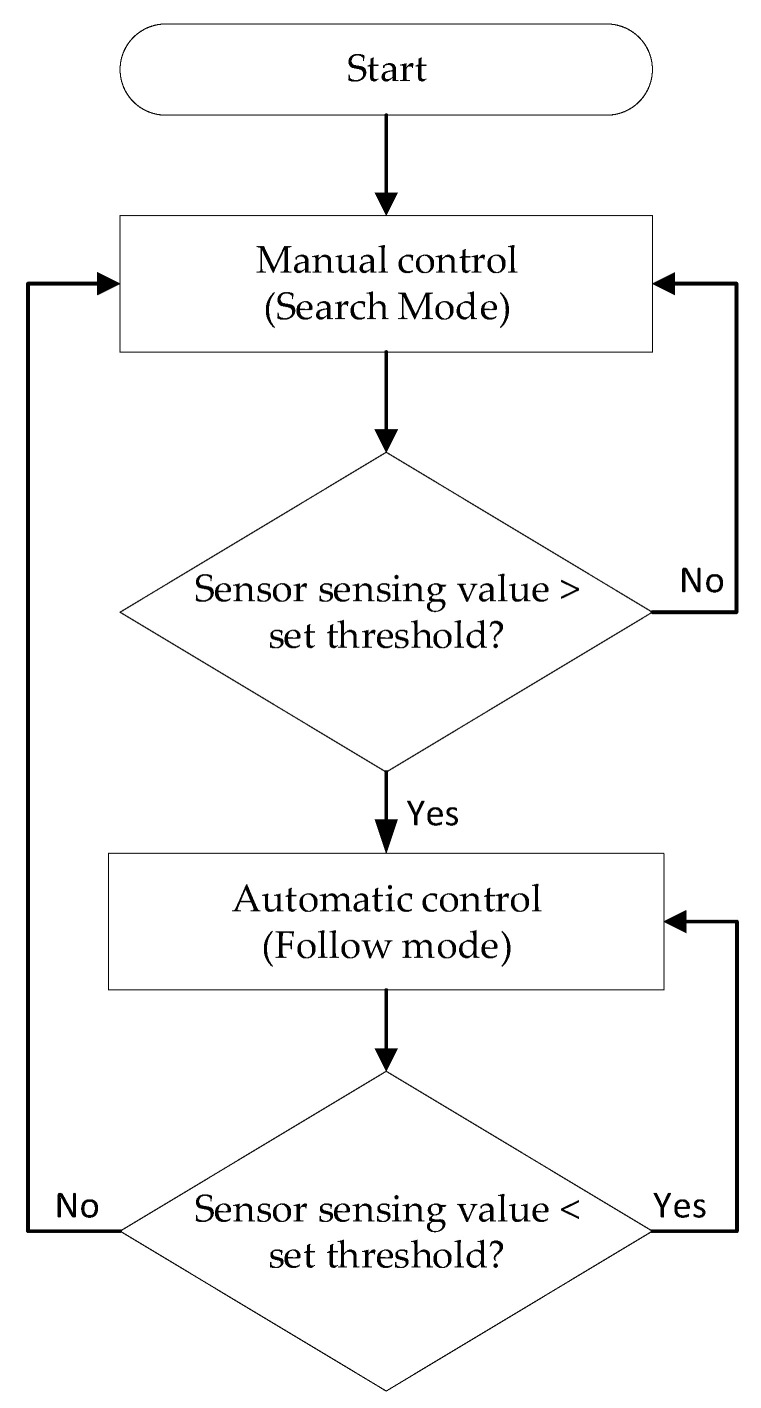
UAV control flow chart.

When the UAV is far from the transmission line, it cannot realize the transmission line inspection according to the collected electric field value, and it needs to control the UAV flight manually. Therefore, the following control plan is formulated. The control flow chart is shown in Figure 7.

The above flow chart is summarized as: (1) The operator controls the UAV while it is not yet close to the transmission line; (2) when the UAV is close to the transmission line and the value sensed by the electric field sensor is greater than the set threshold, it means that the UAV is already in the vicinity of the transmission line and the flight mode is changed at this time; (3) switch to automatic control mode to control the UAV flight based on the electric field value detected by the sensor array; and (4) when the value sensed by the electric field sensor is less than the set threshold, it switches to manual control mode.

## 3. Three-Phase Transmission Line Method Validation

The above theoretical derivation process is based on the case of a single transmission line. However, the transmission line inspected by the UAV is a three-phase, three-wire system, and the electric field distribution of a three-phase transmission line is more complex compared to that of a single-phase transmission line. Therefore, in order to further verify the feasibility of the method in the three-phase transmission line scenario, the electric field distribution of the three-phase transmission line is analyzed.

### 3.1. Three-Phase Transmission Line UAV Flight Area Analysis

For the array to be able to achieve accurate positioning of the transmission line, there cannot be a situation where the same electric field strength exists at different locations, and the relationship between the electric field strength and the distance from the Transmission Lines in the area where the UAV is located should be monotonic. Therefore, the flight area of the UAV needs to be determined based on the electric field distribution of the three-phase transmission line.

It is known from the reference [15] that the maximum electric field strength that the UAV can withstand is 50 kV/m and exceeding this threshold will affect the flight of the UAV. However, in the 10-kV transmission line scenario, this electric field strength is only reached at a distance of 5 cm from the transmission line. Therefore, in order not to consider the impact of the electric field strength around the transmission line on the UAV flight, a 10-kV three-phase transmission line is chosen as the object for analysis.

**Figure 8:**
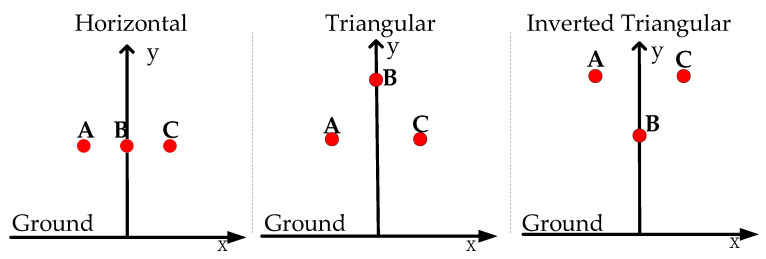
Different arrangements of three-phase transmission lines. A: A-phase transmission line; B: B-phase transmission line; C:C-phase transmission line.

Three-phase transmission lines have different arrangements. The 10-kV three-phase transmission lines are arranged in the following ways: horizontal arrangement, triangular arrangement, and inverted triangular arrangement, as shown in Figure 8 below.

The electric field distribution of different arrangements of transmission lines is different. Therefore, in this paper, the electric field distribution was simulated using COMSOL for different arrangements and, according to the contents of the Reference [21], the relevant parameters of 10-kV transmission lines were determined, as shown in Table 1 below.

The voltages of phase A, phase B, and phase C satisfy the following relationship.

(19) $$\left\{ \begin{array}{l} {\overset{\cdot }{U}}_{A}=\frac{U}{\sqrt{3}}\\ \overset{\cdot }{U_{B}}=\frac{U\cos(-2\pi /3)}{\sqrt{3}}+j\frac{U\sin(-2\pi /3)}{\sqrt{3}}\\ \overset{\cdot }{U_{C}}=\frac{U\cos(2\pi /3)}{\sqrt{3}}+j\frac{U\sin(2\pi /3)}{\sqrt{3}} \end{array}\right.$$

where *U* = 10 kV.

**Figure 9:**
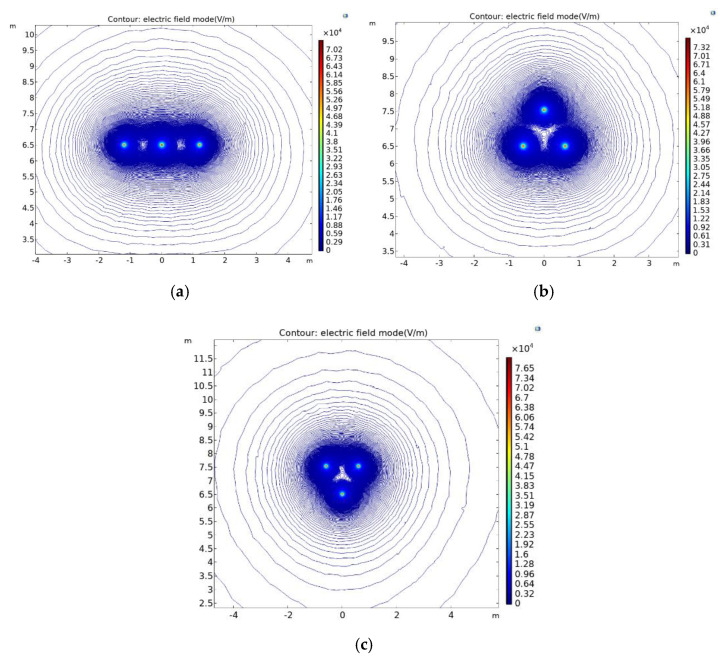
Electric field distribution in different arrangements: (**a**) Horizontally arranged electric field distribution; (**b**) Triangularly arranged electric field distribution;(**c**) Inverted triangular arrangement of the electric field distribution.

The results of the electric field distribution for the three different arrangements are shown in Figure 9.

**Figure 10:**
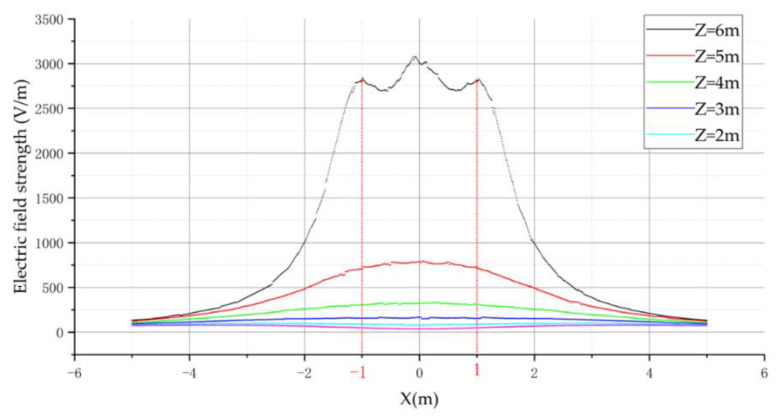
Electric field distribution curve of horizontal arrangement.

From the above figure, we can see that for the electric field distribution of different arrangements of 10 kV, the basic rule is that the further away from the transmission conductor, the smaller is the electric field strength. At the same time, the electric field vectors generated by the three-phase conductor will be superimposed on each other, thus leading to an increase in the field strength around the variable-phase conductor. In order to observe more clearly the field strength distribution of different arrangements, the field strength distribution curves of different arrangements are drawn. The curves for the horizontal arrangement are shown in Figure 10 below, where Z is the height from the ground.

The above electric field distribution curve shows that the three-phase transmission line with horizontal arrangement was symmetrically distributed on the left and right sides with the center-ward conductor as the symmetry axis. However, the variation of electric field intensity on the side of the center-directed wire was not completely monotonic, and the variation of electric field intensity satisfied the monotonic relationship in the regions of X < −1 and X > 1. Therefore, for the horizontal arrangement, the flight area of the UAV is the left side of the A-phase transmission line or the right side of the C-phase transmission line.

**Figure 11:**
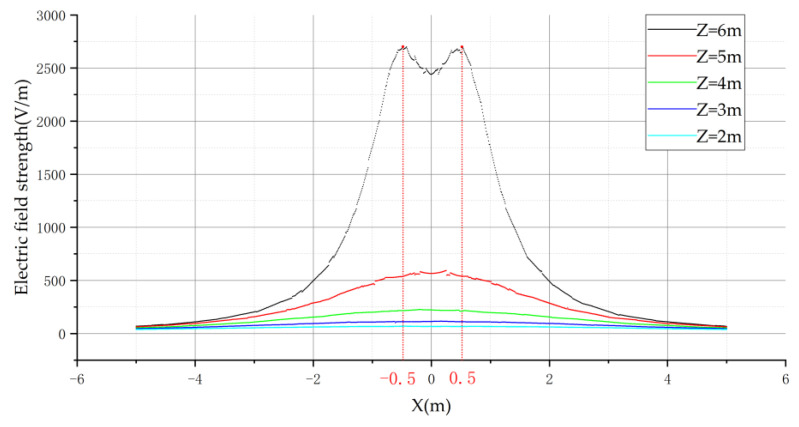
Electric field distribution in triangular arrangement.

In further analysis of the electric field distribution for the triangular arrangement, the electric field distribution curve is shown in Figure 11 below.

The three-phase transmission line in triangular arrangement is also symmetrically distributed on the left and right axis of symmetry with the center wire, and the electric field distribution of the horizontal arrangement is the same in the center wire side of the electric field variation is not monotonic. In the region of X < −0.5 and X > 0.5, the variation of electric field intensity satisfies monotonicity. Therefore, for the triangular arrangement, the flight area of the UAV is equally to the left of the A-phase transmission line or to the right of the C-phase transmission line.

**Figure 12:**
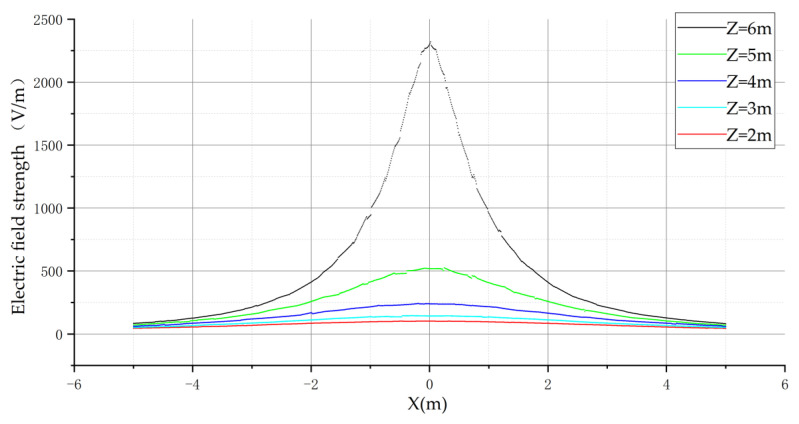
Electric field distribution in inverted triangular arrangement.

Finally, the electric field distribution of the transmission line in the inverted triangular arrangement was analyzed and the curves obtained are shown in Figure 12 below.

The electric field distribution law of the transmission line in the inverted triangle arrangement is similar to that of the remaining two arrangements, both of which have the medium-phase conductor as the symmetry axis. The electric field is symmetrically distributed on the left and right sides of the medium-phase conductor, and the electric field variation on the left and right sides of the medium-phase conductor is monotonic. Therefore, for the inverted triangle arrangement, the flight area of the UAV is the left or right side of the B-phase transmission line.

### 3.2. Analysis of the Electric Field in the Flight Area of the UAV

According to the theoretical derivation process in Section 2, it is known that the electric field sensor array can achieve the localization of transmission lines based on the fundamental principle that the electric field strength at a point around a single-phase transmission line is inversely proportional to the distance from the transmission line. Therefore, it is necessary to verify whether the electric field distribution law in the UAV flight area is consistent with the electric field distribution law of the single-phase transmission line.

**Figure 13:**
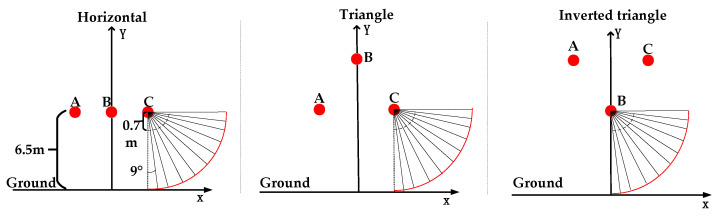
The flight area is divided into equal intervals. A: A-phase transmission line; B: B-phase transmission line; C:C-phase transmission line.

Through the above simulation analysis, we can know that the electric field distribution under the three different arrangements is symmetrically distributed in the left and right direction with the mid-directional wire as the symmetry axis. Therefore, the right part of the UAV flight area is selected for analysis, and the simulation model schematic shown in Figure 13 was constructed as follows.

**Figure 14:**
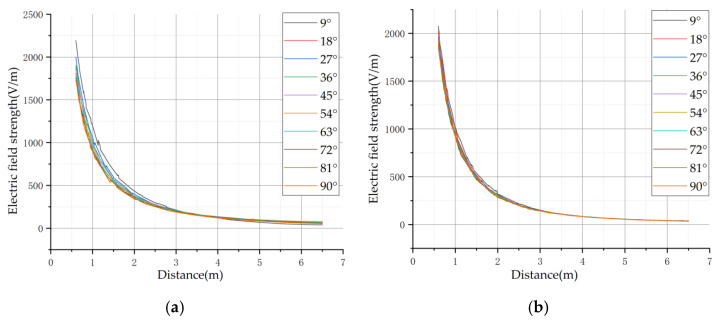

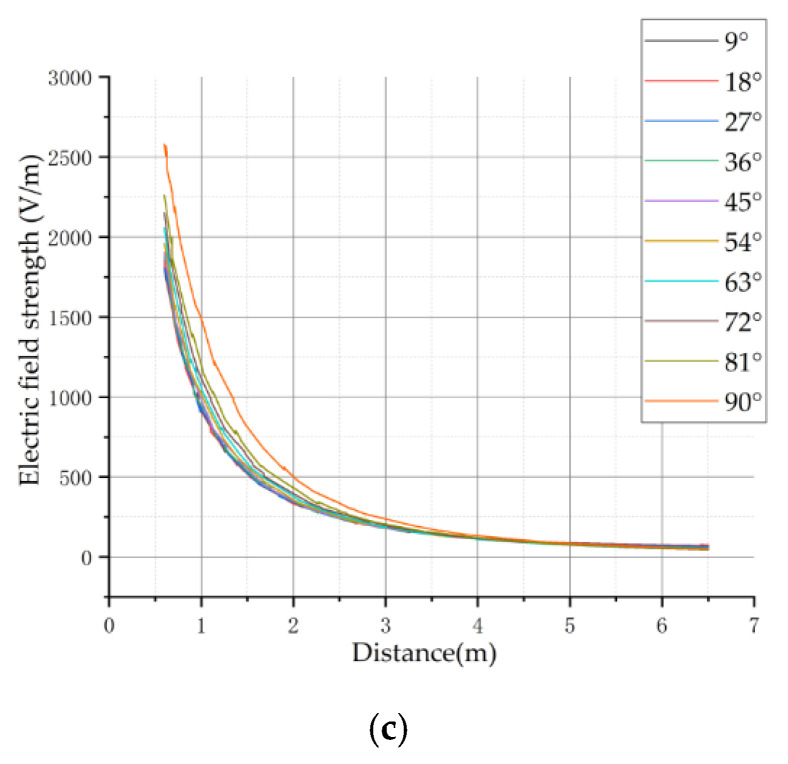
Electric field strength versus distance curves in the flight area of UAVs of different arrangements: (**a**) Horizontal arrangement; (**b**) Triangular arrangement; (**c**) Inverted triangular arrangement.

To avoid the UAV being too close to the transmission line, resulting in impact to the transmission line, the area outside the 1/4 circle with the dashed line in the figure was selected as the UAV flight area. The electric field distribution in the area was analyzed. The following Figure 14 is the electric field strength versus distance for each straight line in the flight area of the three arrangements.

Before analyzing the resulting curves, the concept of goodness of fit was used. The goodness of fit is the degree to which the regression line fits the observed values and is also referred to as the coefficient of determination, expressed as $R^{2}$.

The closer the value of $R^{2}$ is to 1, the better the fit of the regression line to the observed values, and vice versa for the worse the fit of the regression line to the observed values. Then, $R^{2}$ can also be used to characterize the fit of the two curves. The closer $R^{2}$ is to 1, the better the fit of the two curves, and vice versa for the lower the fit. The formula for calculating $R^{2}$ is shown below.

(20) $$R^{2}=1-\frac{\sum_{i}{\left(y_{i}-f_{i}\right)}^{2}}{\sum_{i}{\left(y_{i}-\hat{y}\right)}^{2}}$$

The coincidence of the two curves is measured by $R^{2}$, where $y_{i}$ is the value of the A curve at point *i*, $f_{i}$ is the value of the B curve at point *i*, and $\hat{y}$ is the average of all points of the A curve.

For the curves of electric field strength and distance in horizontal arrangement, the $R^{2}$ of the two curves with the lowest coincidence was 0.9185. For the curves of electric field strength and distance in triangular arrangement, the $R^{2}$ of the two curves with the lowest coincidence was 0.9883. For the curves of electric field strength and distance in inverted triangular arrangement, the $R^{2}$ of the two curves with the lowest coincidence is 0.8137. Based on the derived $R^{2}$, it can be known that the electric field distribution pattern can be considered consistent in the UAV flight area. The reason for calculating $R^{2}$ for the two curves with the lowest overlap is to verify that the electric field distribution pattern is consistent in the flight region specified in Figure 12. If the electric field distribution pattern is not consistent, it is not possible to use the same method to locate the transmission line in that region.

**Figure 15:**
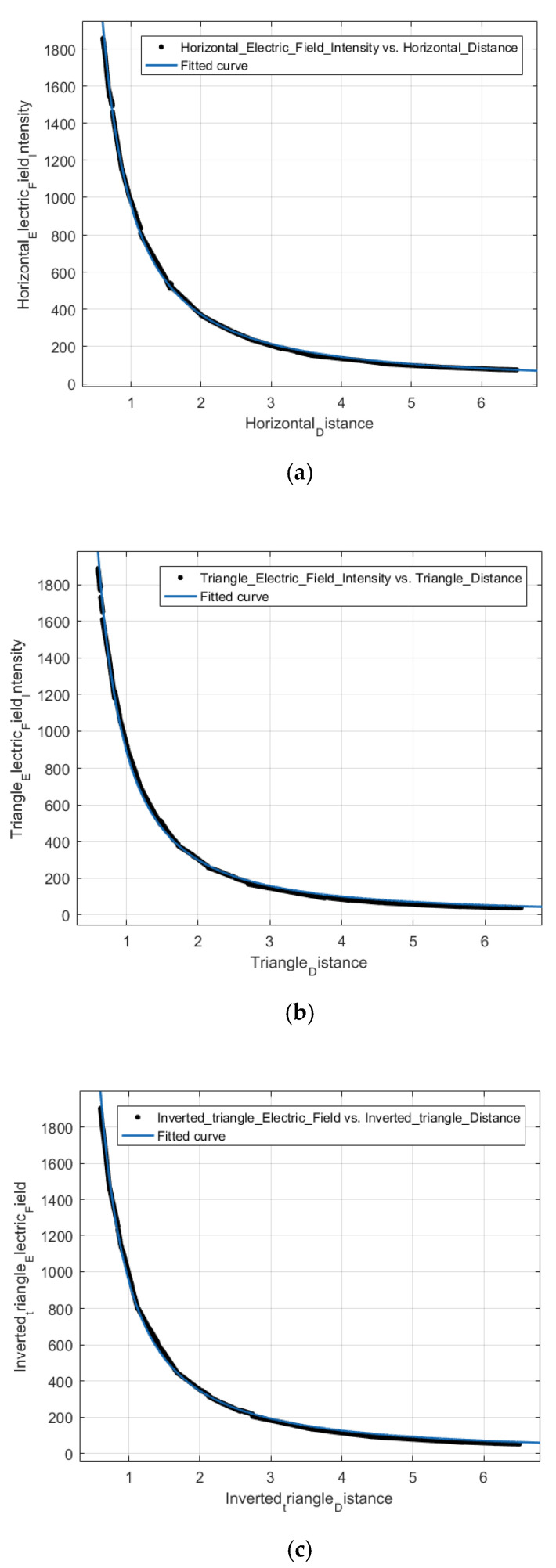
Fitting curves of electric field strength and distance for different arrangements: (**a**) Horizontal arrangement; (**b**) Triangular arrangement; (**c**) Inverted triangular arrangement.

Further, to analyze the relationship between the electric field strength in the UAV flight region and the distance from the Transmission Lines, the average of 10 sets of data in the UAV flight region of Figure 14 was selected for fitting. The fitted curves and equations are shown in Figure 15, divided into three cases, namely, horizontal alignment, triangular alignment, and inverted triangular alignment.

The fitted equations for the three different arrangements and the corresponding $R^{2}$ are shown in Table 2 below.

It can be seen that the electric field strength characterized by the fitted function was not inversely proportional to the distance in the UAV flight region. To investigate whether it can be equated to an inverse proportional relationship, the $R^{2}$ of the fitting function and the inverse proportional function were calculated in the range of ρ ϵ (0.7,6.5) in the UAV flight. The results are shown in Table 3, where the magnitude coefficients of the primary inverse proportional function are equal to the magnitude coefficients of the fitted function.

It can be known by $R^{2}$ that the relationship between electric field strength and distance in the flight area of the three-phase transmission line UAV can be equated as an inverse proportional relationship, and the electric field strength in this area can be considered as the same as that of the single-phase transmission line. Therefore, it can also be shown that the transmission line tracking and localization method proposed in this paper can be used in a three-phase transmission line scenario.

## 4. Research on Sensors and Arrays

### 4.1. Electric Field Sensor and Hardware System Design

**Figure 16:**
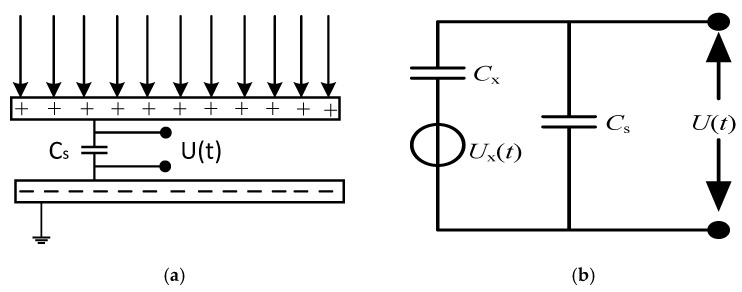
Electric field sensor: (**a**) Sensor schematic; (**b**) Equivalent circuit diagram.

In order to experimentally verify the feasibility of the method, a parallel plate electric field sensor and a signal conditioning circuit were designed. Figure 16 shows the schematic diagram of the parallel plate electric field sensor and the corresponding equivalent circuit diagram.

In Figure 16a, the upper and lower pole plates are connected to the two ends of the sampling capacitor Cs. The voltage signal generated by the induced charge of the pole plates on the sampling capacitor Cs was used as the output signal, the magnitude of which is related to:

(21) $$U(t)=Q(t)/C_{s}$$

It is known from Gauss’s theorem that there was an induced charge generated on the metal pole plate in the electric field E. The surface density of the induced charge is σ, where ε is the dielectric constant of the medium between the pole plates. The change in the number of induced charges caused by the change in the measured electric field strength is:

(22) Q(t)=∫σds=εE(t)S

where *Q(t)* is the induced charge of the pole plate, *E(t)* is the measured electric field strength, and *S* is the effective area of the induced pole plate [22,23].

According to Equations (21) and (22), it is obtained that:

(23) $$U(t)=(\varepsilon E(t)S)/C_{s}$$

Equation (23) shows that the electric field intensity at the measurement location can be obtained by collecting the voltage across the sampling capacitor *Cs*.

**Figure 17:**
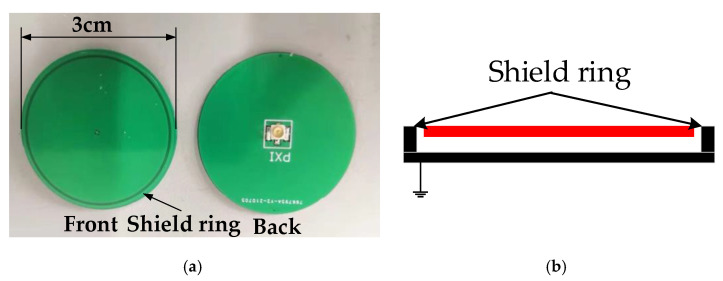
Electric field sensor: (**a**) Physical view of the sensor; (**b**) Schematic diagram of the sensor structure.

To improve the measurement accuracy, in this paper, an electric field sensor with a shielded ring was designed, as shown in Figure 17a according to the reference [10], thus weakening the mutual interference between sensors in the sensor array. In Figure 17b, the front side is the upper pole plate of the parallel plate capacitor and the outermost circle of the front side is the shielding ring; the reverse side is the lower pole plate of the parallel plate capacitor. Figure 17b is a schematic diagram of the structure of the electric field sensor. It can be seen that the shielding ring was connected to the lower pole plate.

**Figure 18:**
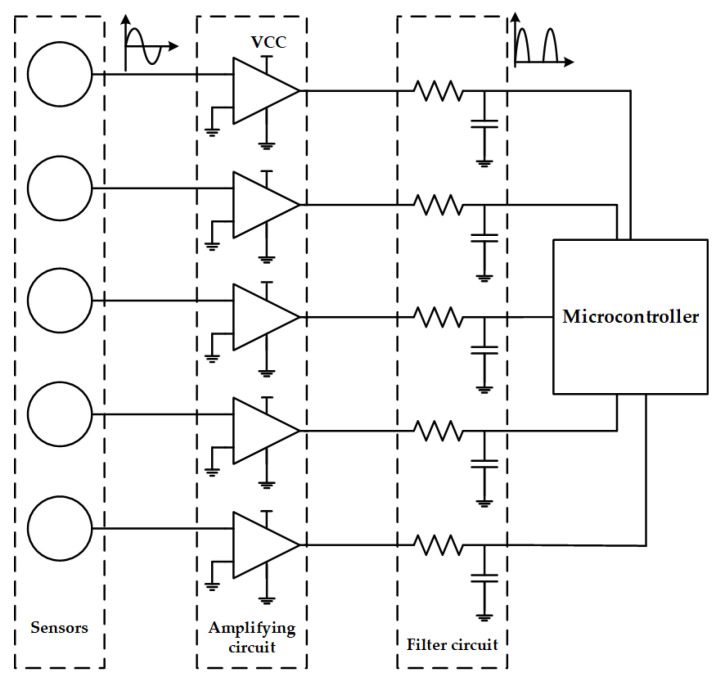
Hardware system schematic.

Figure 18 shows a schematic of the hardware system of the sensor array, which consisted of an electric field sensor, an amplifier circuit, a filter circuit, and a microcontroller. The op-amp in the figure was powered by a single power supply, so the final input signal to the microcontroller was a half-wave signal that was always in the positive half-axis.

**Figure 19:**
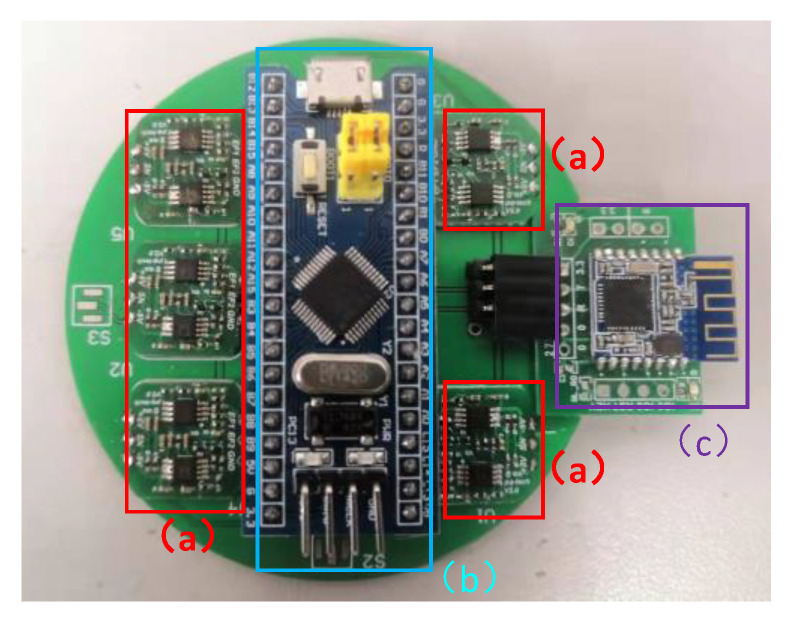
Hardware system physical diagram. (a) signal conditioning circuit; (b) STM32 minimal system board; (c) Bluetooth communication module.

Its hardware physical diagram is shown in Figure 19, where (a) represents the signal conditioning circuit, which amplifies and filters the signal collected by the sensor; (b) represents the STM32 minimal system board, which serves as the master control of the current data acquisition system and can run the FFT algorithm to extract the collected data of 50-Hz frequency components; and (c) represents the Bluetooth communication module, which transmits the data to the PC for analysis by wireless transmission.

### 4.2. System Real-Time Analysis

When controlling the flight of the UAV, the collected information needs to be processed in real time. If the data processing time is too long, the real-time control of the UAV cannot be achieved. For electric field information processing, the frequency component at 50 Hz needs to be extracted, and FFT processing is required. The FFT algorithm uses ST’s assembly library for signal processing, and the Table 4 shows the execution time of the assembly library at different main frequencies and different sampling points.

The current microcontroller used was STM32F103C8T6 with a 72-MHz main frequency and a set frequency resolution of 50 Hz, using a 64-point FFT with a sampling rate of 3.2 KHz. When using STM32 for sampling, use TIM (timer) to trigger AD acquisition and the DMA (direct memory access) mechanism to synchronize the acquisition of five channels of signals. Therefore, the time spent to finish collecting 64 points of data and processing was 64 × 1/3200 + 0.078 ms × 5 = 20.38 ms and the speed of the UAV when tracking transmission lines was generally 20~40 km/h [2]. Therefore, the time spent on current data processing met the requirements of UAV real time.

### 4.3. Sensor Array Detection Area Analysis

To enable the tracking and localization of transmission lines in the flight area of the UAV, the sensor array needs to sense this change when the position of the UAV changes. Therefore, the detectable area of the array was studied based on the resolution of the electric field sensors and the spacing of two adjacent sensors in the array.

The two conditions in which the sensor array can sense the position change of the UAV are:

1. The field strength at the location of the array is higher than the minimum value of the field strength that can be detected by the sensor.
2. The difference in electric field strength between g1 and g0 or g3 and g0 (two sensors perpendicular to the transmission line when flying along a straight wire) should be greater than the minimum resolution of the field strength of the sensor.

The first condition satisfies the resolution requirement of a single sensor, and the second condition satisfies the resolution requirement of a sensor array. It can be seen that, as long as the second condition is satisfied, the first condition can also be satisfied.

Through experimental verification, the field strength resolution of the electric field sensor used was 10 V/m. Therefore, it was necessary to ensure that the difference between the electric field strength of g1 and g0 or g3 and g0 was greater than or equal to 10 V/m. The radius of the sensor array was set to 20 cm for the current sensor array with reference to the rotor size of the DJI Genie 4Pro series UAV.

**Figure 20:**
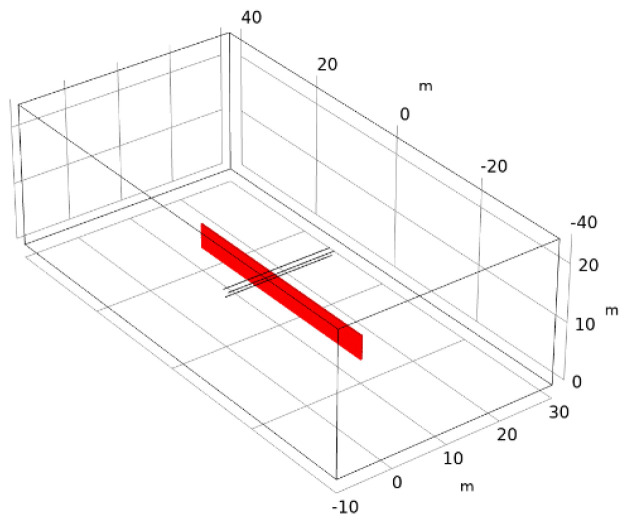
Field strength point scan range.

The field strength was scanned in the area below the transmission line, and the field strength data were exported at three sensor locations in space (two adjacent sensors perpendicular to the transmission line). The red area of Figure 20 shows the scanning range of the field intensity in the 10-kV electric field and the scanning of points along the y, z coordinate axes in the cross section of x = 10 m with a scanning interval of 0.2 m, Detailed parameters are shown in Table 5.

**Figure 21:**
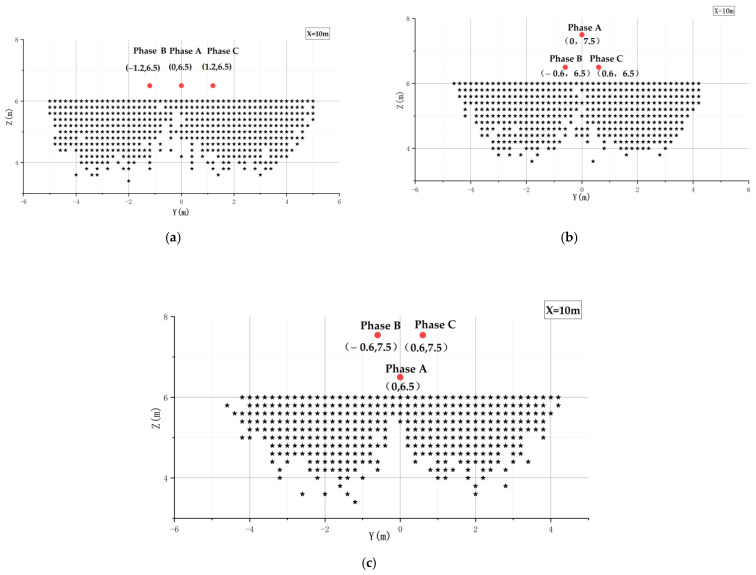
Sensor array measurement range diagram for different arrangements: (**a**) Horizontal arrangement; (**b**) Triangular arrangement; (**c**) Inverted triangular arrangement.

Based on the data obtained from the scan, the point where the difference between two adjacent sensors perpendicular to the transmission line is greater than 10 V/m is called the effective point, which is the area where the electric field sensor array can sense the change in electric field. The valid points are represented using a scatter plot, as in Figure 21, where the red dots are the locations of the transmission lines and the black dot areas are the valid point areas.

As can be seen from Figure 21, the range of effective points for different arrangements of transmission lines was also different. The superposition of the field strength of the three-phase transmission lines led to a smaller range of effective points below the mid-phase transmission lines, while the effective points were mainly concentrated on the side of the side-phase transmission lines.

It can be seen that the UAV flight area determined in Section 3.1 was included in the detection area of the sensor array, indicating that the sensor array was able to sense this change when the position of the UAV changed in the flight area.

Finally, the final UAV flight area was determined based on the detectable area of the sensors and the UAV flight area derived in Section 3.1, as shown in Table 6.

## 5. Experimental Verification

**Figure 22:**
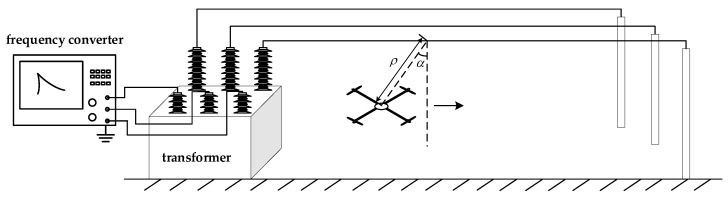
Schematic diagram of the experimental platform.

**Figure 23:**
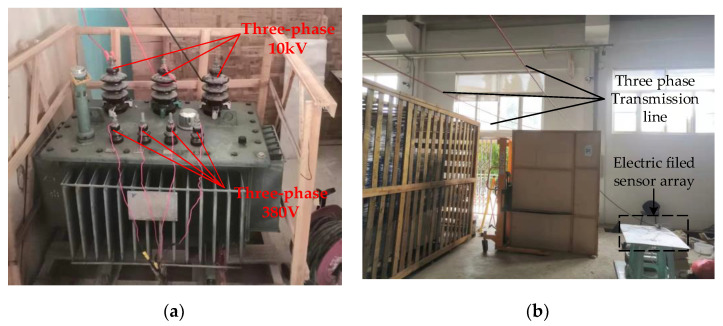
Physical diagram of the experimental platform. (**a**) 10kV transformer physical figure; (**b**) Experimental site figure.

In the laboratory environment, a 10-kV, three-phase transmission line experimental platform was built, where the sensor array was located at the lower right corner of the transmission line, the distance of the central sensor g0 from the transmission line ρ = 1 m, and the elevation angle of the transmission line relative to the sensor array α = 45°. In order to verify the correctness of the results by multiple sets of data, the sensor array was moved parallel along the transmission line, keeping ρ and α constant, the values of one set of data were recorded every 40 cm, and a total of 10 sets were recorded. Figure 22 is a schematic diagram of the experimental platform, and Figure 23 is a physical diagram of the experimental platform.

**Figure 24:**
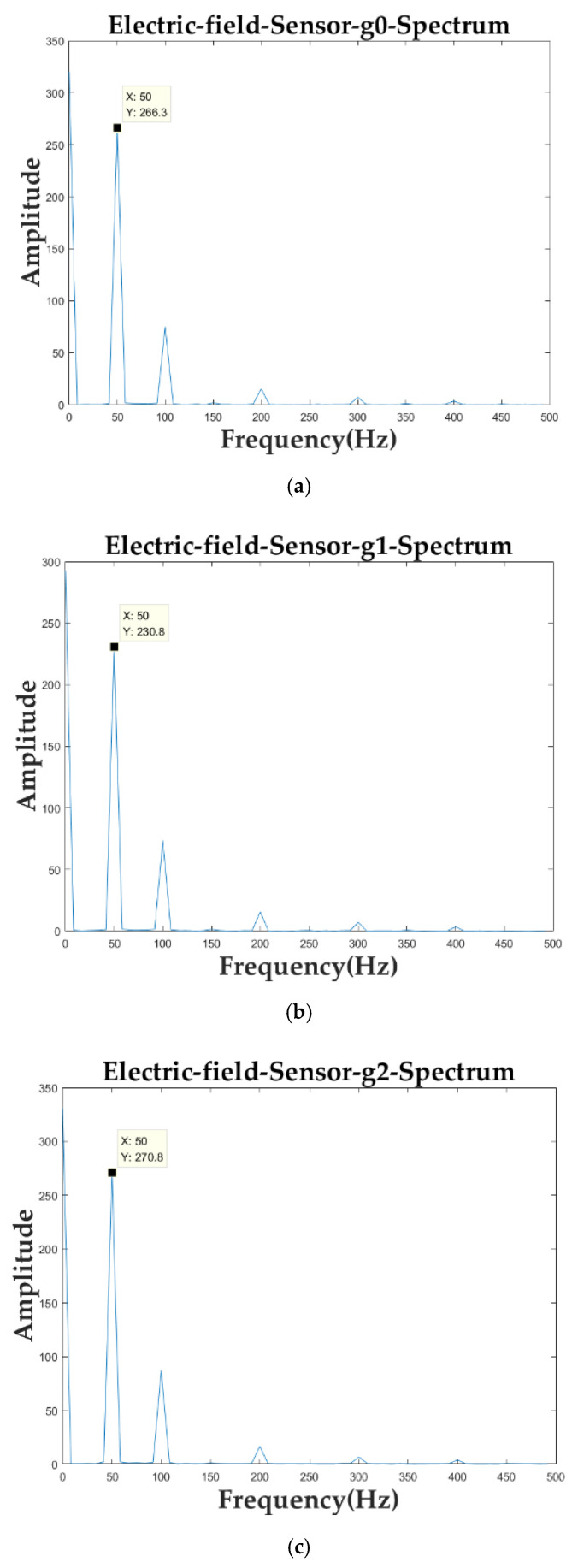

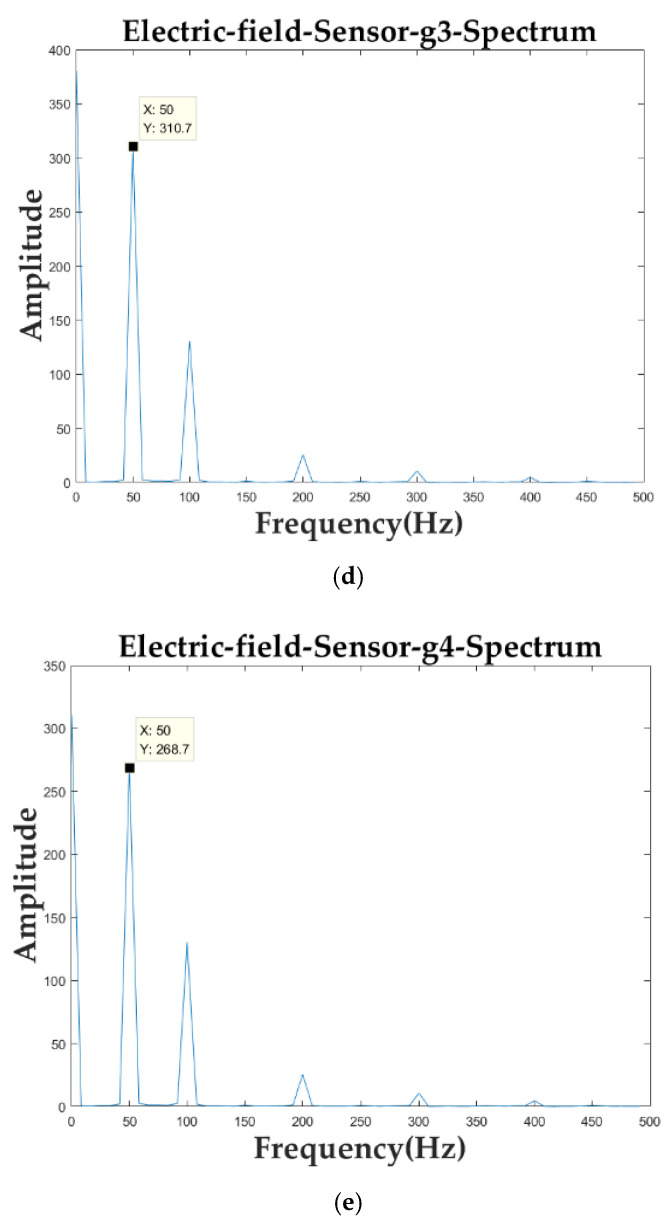
Sensor array data spectrogram. (**a**) Spectrum of sensor g0; (**b**) Spectrum of sensor g1; (**c**) Spectrum of sensor g2; (**d**) Spectrum of sensor g3; (**e**) Spectrum of sensor g4.

The collected data from each sensor were subjected to FFT processing. Figure 24 shows the spectrogram of one of the 10 sets of data from the sensor array.

As can be seen from the above spectrum, the values of the three sensors, g2, g0, and g4, in the sensor array parallel to the transmission line did not differ much in the 50-Hz frequency component, and the values of the three sensors perpendicular to the transmission line in the 50-Hz frequency component were smaller as the distance from the transmission line was farther.

**Figure 25:**
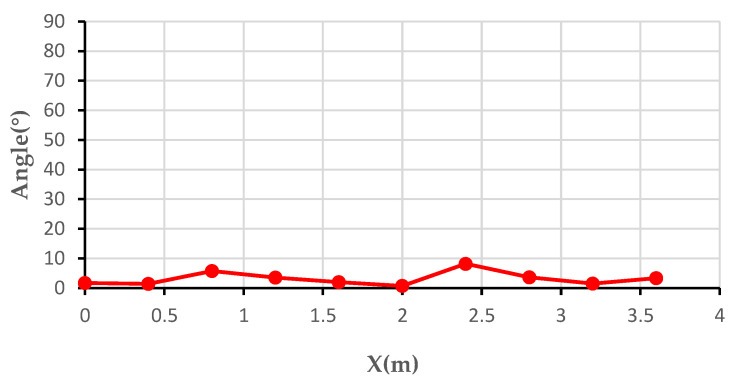
Curve of deflection size of heading angle.

The data at the 50-Hz frequency component of each sensor in the 10 sets of data were extracted separately. The deflection angle of the heading angle was calculated according to Equation (18). Figure 25 shows the graph of the deflection angle of the heading angle.

As can be seen from the above graph, because it was moving along a straight wire, ideally, the calculated heading angle deflection angle should always be 0. However, the actual situation deviated from the ideal situation, and the maximum absolute error is 8.2° in the above graph.

**Figure 26:**
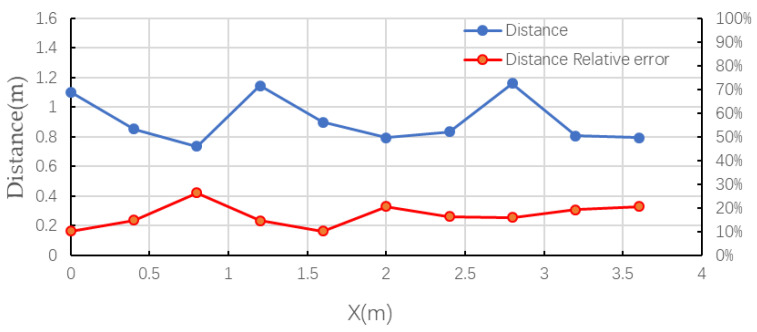
Sensor array to transmission line distance curve.

The distance ρ between the sensor array and the transmission line was calculated according to Equation (15). Figure 26 below shows the distance profile of the sensor array and the transmission line calculated at 10 different locations. 

As can be seen from the above graph, the maximum relative error of the 10 sets of distance measurements was calculated to be 26.3% and the maximum absolute error was 26.3 cm.

**Figure 27:**
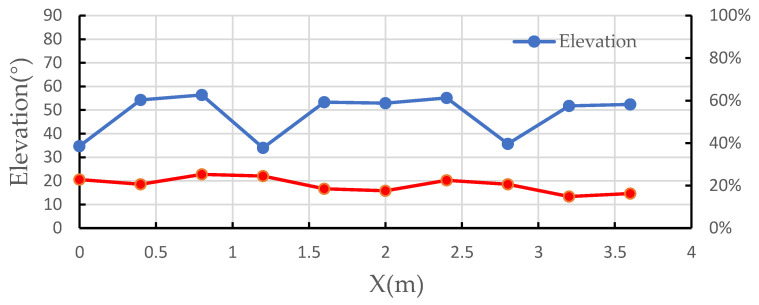
Elevation curve of the transmission line relative to the sensor array.

The elevation angle α of the transmission line relative to the sensor array was calculated according to Equations (1)–(17). Figure 27 shows the calculation results of the elevation angle.

As can be seen from the above graph, the maximum relative error of the 10 sets of measurements was 25.27% and the maximum absolute error was 11.37°.

The above calculation results show that there were some errors between the values calculated by the electric field sensor and the real values. For the UAV heading angle deflection size, the reason for the error mainly came from the interference of the external environment, and the subsequent work can introduce filtering algorithms to reduce the error. For distance error, the application scenario of UAV positioning on transmission lines was mainly used for inspection of transmission lines. One of the main purposes of measuring the distance is to prevent the UAV from hitting the transmission lines, and, in the process of distance judgment, a certain distance margin can be left to avoid the consequences of hitting the transmission lines due to measurement error. For the elevation error, the elevation angle is to enable the UAV to control the camera on the transmission line during the inspection to determine the location of the transmission line fault. The lenses used by UAV cameras can be roughly divided into standard lenses and wide-angle lenses, with standard lenses having a perspective of about 50° and wide-angle lenses having a perspective of 90° or more. Therefore, with the current error in the elevation angle calculation, the UAV was able to complete the inspection of the condition of the transmission line. In summary, it can be shown that the error was within the acceptance range.

The method proposed in this paper is more adaptable to the environment than using machine vision for transmission line inspection and can inspect transmission lines in foggy weather with unclear vision. Compared with using LiDAR to extract 3D point cloud data, it has the advantages of smaller data volume, lower processor load, and higher detection efficiency. The method proposed in this paper also has a great advantage in terms of cost compared to using RTK techniques that require the construction of base stations. On the other hand, the method proposed in this paper also showed some advantages compared with other methods of navigation and localization based on electric fields. In contrast to the position deviation calculation based on electric field information proposed in reference [11], which requires advance knowledge of the electric field value of the preset path, the method proposed in this paper performs the position calculation based on the sensor data in real time. The distance measurement method of UAV and transmission line proposed in reference [13] is based on the measured electric field fitted to the electric field strength as a function of distance, but the function relationship in different directions of the transmission line may be different. The method proposed in this paper divides the flight area of the UAV to be more rigorous. The UAV navigation method proposed in reference [17] navigates according to the electric field distribution characteristics of the medium-phase conductors of three-phase transmission lines, which stipulates that the UAV can only inspect in the vertical direction of the medium-phase conductors. The flight area of the UAV in the method proposed in this paper is somewhat wider.

Therefore, this method, as a new solution for transmission line tracking and positioning, can be applied to UAVs to achieve autonomous inspection.

## 6. Conclusions

(1) This paper built an electric field sensor array according to the electric field distribution law of single-phase transmission lines; calculated the deflection size of the current heading angle of the UAV, the distance of the transmission line relative to the UAV and the elevation angle; and then realized the tracking and positioning of the UAV on the transmission line.
(2) By analyzing the electric field distribution under three different arrangements of 10-kV transmission lines, horizontal, triangular, and inverted triangular arrangements, the flight area of the UAV was determined. The electric field distribution law in the flight area of the UAV was further studied, and it was concluded that the electric field distribution law in the flight area of the UAV can be equated with that of the single-phase transmission line, which verified that the current method can be used in the three-phase transmission line scenario.
(3) A parallel plate electric field sensor with a shielded ring and a signal conditioning circuit were designed. According to the field strength resolution of the electric field sensor array, the detection area of the sensor array with three different arrangements was obtained, which illustrated that the sensor array can sense the change of the UAV position in the UAV flight area.
(4) A 10-kV, three-phase transmission line experimental platform was built. The experimental data showed that when the sensor array moved along the transmission straight wire, the maximum absolute error of the calculated deflection size of the heading angle was 8.2°, the maximum absolute error of the distance between the sensor array and the transmission line was 26.3 cm, and the maximum absolute error of the elevation angle was 11.37°, all of which were within the acceptable range and could be used for the UAV to achieve autonomous inspection.

The transmission line studied in this paper was only for straight conductors, without considering the transmission line bend. In the actual 10-kV transmission line, there are also poles and trees on the field strength distribution. The effect of the sensor metal pole plate as well as the UAV on the electric field distortion has also not been studied in depth. Therefore, these influencing factors will be investigated in subsequent work to further optimize the transmission line tracking and positioning model.

## Figures and Tables

**Table 1:** Transmission line model parameters.

Voltage U	Phase Spacing	Length of Lead Wire	Height above Ground
10 kV	1.2 m	20 m	6.5 m

**Table 2:** Fitting function and *R^2^* results.

Arrangement	Fitting Function	*R^2^*
Horizontal arrangement	$E=908\rho^{-1.382}$	0.9989
Triangular arrangement	$E=893.6\rho^{-1.479}$	0.9975
Inverted triangular arrangement	$E=1018\rho^{-1.455}$	0.997

**Table 3:** Comparison results between the fitted function and the inverse proportional function.

Arrangement	Fitting Function	Inverse Proportional Function	$R^{2}$
Horizontal arrangement ρ∈(0.7,6.5)	$E=908\rho^{-1.382}$	$E=908\rho^{-1}$	0.8942
Triangular arrangement ρ∈(0.7,6.5)	$E=893.6\rho^{-1.479}$	$E=893.6\rho^{-1}$	0.8067
Inverted triangular arrangement ρ∈(0.7,6.5)	$E=1018\rho^{-1.455}$	$E=1018\rho^{-1}$	0.8653

**Table 4:** FFT operation time.

FFT	24 MHz		48 MHz		72 MHz	
	Cycle Count	Time	Cycle Count	Time	Cycle Count	Time
64 points	3847	0.16 ms	4472	0.093 ms	5661	0.078 ms
256 points	21,039	0.876 ms	24,964	0.52 ms	31,527	0.437 ms
1024 points	100,180	4.174 ms	114,350	2.382 ms	153,930	2.138 ms

**Table 5:** Data transmission line around electric field scanning point.

Coordinate Points	Starting Point	Measurement Interval	Ending Point
y(m)	−20	0.2	20
Z(m)	2	0.2	6

**Table 6:** UAV flight area.

Arrangement	Range
Horizontal arrangement	−5 < x < −1.2, 1.2 < x < 5
	4 < y < 5.8
Triangular arrangement	−4.1 < x < −0.6, 0.6 < x < 4.1
	4 < y < 5.8
Inverted triangular arrangement	−4.1 < x < 4.1
	4 < y < 5.8

## Data Availability

The data used to support the findings of this study are included within the article.
